# Comparing Remote Sensing Methods for Monitoring Karst Rocky Desertification at Sub-pixel Scales in a Highly Heterogeneous Karst Region

**DOI:** 10.1038/s41598-019-49730-9

**Published:** 2019-09-16

**Authors:** Xiangkun Qi, Chunhua Zhang, Kelin Wang

**Affiliations:** 1 Key Laboratory for Agro-ecological Processes in Subtropical Region, Institute of Subtropical Agriculture, Chinese Academy of Sciences, Changsha, 410125 China; 2 Huanjiang Observation and Research Station for Karst Ecosystem, Chinese Academy of Sciences, Huanjiang, Hechi 547100 China; 3 Department of Geography and Geology, Algoma University, Sault Ste. Marie, ON P6A2G4 Canada

**Keywords:** Restoration ecology, Environmental impact

## Abstract

Rugged karst terrain relief that creates shadows in satellite imagery, combined with high karst landscape heterogeneity stand in the way of fractional cover retrieval on karst rocky desertification (KRD) monitoring. In this study, we explored the feasibility of applying multispectral high spatial resolution Advanced Land Observing Satellite (ALOS) imagery for the fractional cover extraction of rocky outcrops. Dimidiate pixel model (DPM) and spectral mixture analysis (SMA) approaches (including simple endmember spectral mixture analysis and multiple endmember spectral mixture analysis) were selected to explore their feasibility for KRD monitoring through accuracy improvement for fraction estimation. Results showed fractional cover retrievals at the sub-pixel scale is essential in highly heterogeneous karst landscapes. Indeed, mixed pixels accounted for 93.7% of the study area in southwest China. Multiple endmember spectral mixture analysis achieved high overall accuracy (80.5%) in monitoring the percentage of rocky outcrop land cover. Furthermore, the predicted exposed bedrock coverage via spectral mixture analysis were similar in sunlit and shadow areas for the same surface types. This reflected that SMA methods could effectively reduce topographic effects of satellite imagery to improve the accuracy of fractional cover extraction at sub-pixel level in heterogeneous and rugged landscapes.

## Introduction

Karst landscapes, a special environment formed within carbonate bedrock, are among the most ecologically fragile regions in the world^1,2^. Southwest China has one of the largest continuous karst landscapes on earth; an area of 540 000 km^2,3^. Critically, this karst region has high human population density and limited cropland area^4^. Consequently, the tight human-land relationships have led to subsistence farming over-exploitation and consequently severe environmental problems including karst rocky desertification (KRD). KRD is a progressive process of land degradation in which soil is partially or completely eroded^5^. Results of KRD include exposure of widespread rocky outcrops, declining land productivity, and the formation of desert-like landscapes^6^. As a result, KRD, following soil erosion in the loess plateau and desertification in northwest China, is considered to be one of the most important ecological and environmental problems in China^7^. Therefore, it is necessary to monitor and assess KRD conditions in support of regional sustainable development.

There are various methods available to map KRD. Basic research and qualitative estimates the extent of KRD largely depend on field surveys of vegetation and rocky outcrop cover, slope and soil distribution. These methods are time consuming, expensive and limited by rugged terrain and large spatial scales^8^. Fractional ground cover extracted from remotely sensed images has been widely applied to describe land degradation and human disturbance^9,10^. When KRD occurs, the most obvious land-surface symptoms are low vegetation cover and bedrock exposure. Therefore, the fractional cover of vegetation and exposed rocks are most commonly characterized as land-surface consequences of KRD.

Satellite images have been used to map desertification and its changes over time beginning in late 1990s^11–13^. Commonly used moderate resolution images include Landsat Multispectral Scanner (MSS)^14^ and Landsat Thematic Mapper (TM)^15,16^. These optical satellite images have 30-meter resolutions and are useful for extracting land cover and change conditions at regional scales. However, the large variation in karst landforms (e.g. poljes, valleys, cockpits, towers, and sinkholes) and various degrees of soil erosion create abundant niches for vegetation growth that cause soil discontinuity and vegetation fragmentation^17^. The dissolution of carbonate rocks produces densely distributed ditches on land surfaces that coexist with rocky outcrops, bare soil, grass, shrub and forest within a KRD region^14^. Consequently, it is challenging to identify a pure, rocky spectrum on a relatively fine-scale (e.g. SPOT 10 × 10 m) remote sensed data^18^. Therefore, the high degree of heterogeneity in karst landscapes determines that one pixel in a satellite image often includes more than a single land object.

One feasible solution is to estimate the proportion of land cover at the sub-pixel scale for heterogeneous landscapes^10,19–21^. A dimidiate pixel model (DPM) is commonly used to calculate fractional vegetation cover (FVC) at sub-pixel scales^22^ and this method has been applied in the karst region for KRD monitoring^23,24^. Another widely used method is spectral mixture analysis (SMA)^25^. This approach supposes that reflectance for one pixel is a linear mixture of several endmembers and that each endmember is a unique land cover type with a specific spectral signature. The aim of SMA is to decompose mixed spectra and calculate proportions of each land cover type in a single pixel. The spectral unmixing model has been widely applied to plant species identification^26,27^, fire severity^28,29^ and urban remote sensing^30,31^ with some success. However, there have been few SMA-related studies in the karst region of southwest China.

A critical step in SMA is endmember selection. Unlike the impervious surface of urban areas, bare rocks are often mixed with vegetation and soil. The degree of rocky outcropping has changed due to variation in natural conditions and human disturbance^32^. Furthermore, as high albedo endmembers, rocks, cement road surfaces, building roofs and limestone soils can cause spectral confusion in an image because they have similar, high reflectance. All directly affect the endmember selection of KRD. High spatial resolution imagery (e.g., SPOT-5 and Advanced Land Observing Satellite (ALOS) images) have smaller pixel size that would be conductive to select pure endmembers for spectral mixture analysis compared to medium resolution images (30 × 30 m resolution). High resolution imagery could provide greater detail and capture more spatial variation to help explore mechanisms of KRD dynamics. These all suggest that high spatial resolution imagery is promising for fine-scale karst land applications.

In addition to being highly heterogeneous, the relatively high elevation contrast in the karst area causes significant shadow effects in remotely sensed images. The topographic effect in images is limiting because weak reflectance from shadow areas commonly complicate information extraction^20,33^. NDVI is able to minimize variation in topographic effects on the spectral properties of land surfaces and so is widely used in the DPM for FVC estimations in karst regions^34^. In contrast, SMA treats shade as a separate image component and eliminates the shadow fraction by area redistribution^35^. However, there is no relevant research that proves it can be applied for topographic correction in karst regions.

Our study attempted to take advantage of high resolution optical multispectral ALOS images to extract the fractional cover of rocky outcrops at a sub-pixel level in the karst region of southwest China. We applied and compared the efficacy of DPM and two SMA methods through (1) accuracy evaluation of extracting rocky outcrop fractions, (2) assessment of topographic effects of fractional images in each method, and (3) analyzing the KRD status. Results from this study serve as a technical reference for applying optical remote sensing in heterogeneous and rugged terrain regions.

## Results

### Endmember spectral analysis

**Figure 1:**
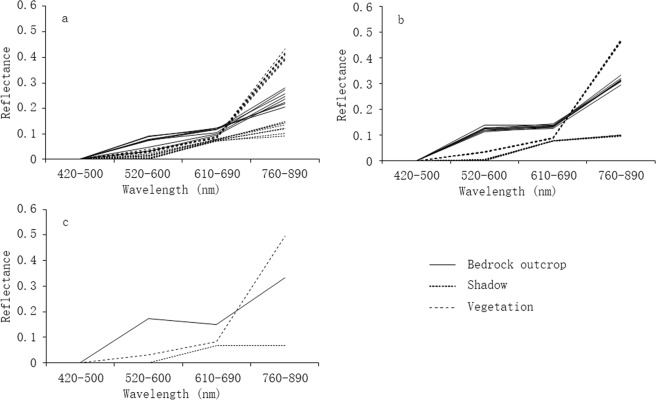
Spectral curves of rocky outcrops, vegetation and shadow in the study area. (**a**) Was collected from ALOS image-based field survey sample sites; (**b**) was obtained based on MESMA; and (**c**) from SESMA.

We built three spectral libraries using ALOS multispectral images based on separate methods (Fig. 1). Eight representative spectral curves for each land type were developed from ALOS images according to field sample sites and MESMA (Fig. 1a,b). Spectral curves in vegetation and shadow classes had similar shapes. Although the spectral measurements data extracted based on field sample sites were deemed as pure endmembers, spectral curves of rocky outcrops had large variation. This appeared to be related to differences in aspect and slope at sample sites causing variation in image spectral signature. In addition, surface characteristic changes (e.g., color) of rocky outcrops affected spectral reflectance creating further uncertainty in representing rocky outcrop characteristics using spectral measurements. Based on MESMA, the eight representative spectral curves of each land type were developed using the lowest Root Mean Square Error (RMSE) within each class. Spectral curves within each class were similar, although the curves of rocky outcrops did vary (Fig. 1b). This indicates that the spectral signature of rocky outcrops may also vary.

One representative spectral curve from each class was developed using SESMA (Fig. 1c) and spectral curves of three land types were determined separately. The spectral curve with high reflectance in the 520–600 nm and 610–690 nm bands represents the characteristics of rocky outcrops. Comparing these three methods, the spectra of representative endmembers measured at field sample sites was inconsistent and difficult to use to represent the features of rocky outcrops. Therefore, the spectral selections from MESMA and SESMA were used to extract rocky outcrop coverage.

### Accuracy assessment of rocky cover extraction

Fraction image accuracy from MESMA, SESMA and DPM were calibrated using validation points (see methods). The best prediction was achieved using MESMA (Table 1). The overall classification accuracy of the whole study area (based on MESMA) was 76.4%, significantly higher than that from SESMA (50.8%) and DPM (54.2%). The Kappa coefficient from MESMA was slightly lower (0.705). Classification accuracy of rocky outcrop estimates was higher in sunlit areas than in shadow areas. MESMA acquired the highest accuracy in sunlit areas (83.7%) and shadow areas (60.4%). In contrast, the fractional cover of rocky outcrops from SESMA had lower accuracy in sunlit areas (51.3%). Coverage from DPM had very low accuracy in shadow (18.2%).

**Table 1:** Overall accuracy (OA), and Kappa coefficient (K) of percentage of estimated rocky outcrops.

	Sunlit area			Shadow area			All study area		
	MESMA	SESMA	DPM	MESMA	SESMA	DPM	MESMA	SESMA	DPM
OA (%)	83.7	51.3	69.2	60.4	49.7	18.2	76.4	50.8	54.2
K	0.796	0.387	0.614	0.498	0.344	0.021	0.705	0.374	0.429

Error matrixes for sunlit areas were used to explore the estimate error in percentage of rocky outcrops. In sunlit areas, MESMA successfully predicted the cover of bedrock outcrops at each level (Table 2). Producer’s accuracies (PA) were between 0.79 and 0.93. SESMA achieved higher accuracy in the areas with lower bedrock outcrop cover (10–50%). However, accuracies were lower when bedrock outcrop cover exceeded 50% (producer’s accuracies were less than 0.19). Many areas with high bedrock outcrop cover were classified as low bedrock outcrop area, underestimating bedrock cover based on SESMA in sunlit areas. DPM yielded high accuracies in low and high bedrock outcrop cover areas. However, for the bedrock outcrop cover between 10% and 70%, producer’s accuracies were less than 60%, largely because of the underestimation of bedrock outcrop coverage.

**Table 2:** Percentage of rocky outcrops in class confusion matrix in sunlit and shadow areas.

MESMA_sunlit Cover (%)		Classified data					PA
		0–10	10–30	30–50	50–70	70–100	
Reference data	0–10	60	15	1	0	0	0.79
	10–30	0	66	17	0	0	0.80
	30–50	0	1	63	14	0	0.81
	50–70	0	0	0	61	9	0.87
	70–100	0	0	0	5	68	0.93
	UA	1.00	0.80	0.78	0.76	0.88	
**SESMA_sunlit** **Cover (%)**		**Classified data**					**PA**
		**0–10**	**10–30**	**30–50**	**50–70**	**70–100**	
Reference data	0–10	34	42	0	0	0	0.45
	10–30	1	77	5	0	0	0.93
	30–50	0	21	57	0	0	0.73
	50–70	0	0	57	13	0	0.19
	70–100	0	1	21	37	14	0.19
	UA	0.97	0.55	0.41	0.26	1.00	
**DPM_sunlit** **Cover (%)**		**Classified data**					**PA**
		**0–10**	**10–30**	**30–50**	**50–70**	**70–100**	
Reference data	0–10	76	0	0	0	0	1.00
	10–30	33	50	0	0	0	0.60
	30–50	2	47	25	4	0	0.32
	50–70	0	1	27	40	2	0.57
	70–100	0	0	0	1	72	0.99
	UA	0.68	0.51	0.48	0.89	0.97	
**MESMA_shadow** **Cover (%)**		**Classified data**					**PA**
		**0–10**	**10–30**	**30–50**	**50–70**	**70–100**	
Reference data	0–10	10	16	3	0	0	0.34
	10–30	3	25	12	2	0	0.60
	30–50	1	2	23	11	1	0.61
	50–70	0	1	4	17	7	0.59
	70–100	0	0	0	0	21	1.00
	UA	0.71	0.57	0.55	0.57	0.72	
**SESMA_shadow** **Cover (%)**		**Classified data**					**PA**
		**0–10**	**10–30**	**30–50**	**50–70**	**70–100**	
Reference data	0–10	4	25	0	0	0	0.14
	10–30	1	29	12	0	0	0.69
	30–50	2	12	23	1	0	0.61
	50–70	0	3	13	13	0	0.45
	70–100	0	0	2	9	10	0.48
	UA	0.57	0.42	0.46	0.57	1.00	
**DPM_shadow** **Cover (%)**		**Classified data**					**PA**
		**0–10**	**10–30**	**30–50**	**50–70**	**70–100**	
Reference data	0–10	2	12	14	1	0	0.07
	10–30	0	1	5	11	25	0.02
	30–50	0	0	3	7	28	0.08
	50–70	0	0	0	2	27	0.07
	70–100	0	0	0	0	21	1.00
	UA	1.00	0.08	0.14	0.10	0.21	

(PA: producer’s accuracy; UA: user’s accuracy).

The accuracy of rocky outcrops estimation was lower in shadow compared with sunlit areas. Using MESMA, producer’s accuracy ranged between 1.00 and 0.34. A confusion matrix showed that the rocky outcrop cover was somewhat overestimated for each cover class. Although the results from SESMA reached high producer’s accuracies in areas with the rocky outcrop coverage between 10% and 50%, the predicted coverage was consistently underestimated in shadows. Alternatively, DPM clearly overestimated rocky outcrop cover in shadow areas suggesting that it is greatly affected by shade.

### Topographic effects

**Figure 2:**
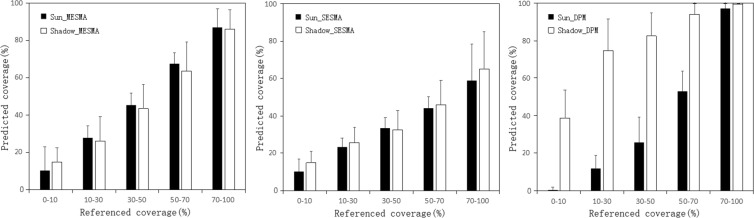
Predicted percent of rocky outcrop in sunlit and shadow areas.

The mean cover of rocky outcrop estimated from MESMA was similar in sunlit and shadow areas in each rocky outcrop category (Fig. 2). Although the cover predicted in shadows was smaller than that in sunlit areas, the largest difference of predicted value in these two areas was not greater than 6.4%.

The predicted value using MESMA also matched the reference data well. Each prediction in sunlit and shadow areas fell into the range of reference data. The estimated percentages of bedrock outcrop estimated by SESMA were also similar in sunlit and shadow areas. However, the predicted cover values were underestimated, especially in areas with large areas of rocky outcrop. For example, in the range from 51% to 70% cover, the prediction was just less than 46.1%. There were large differences in predicted values for sunlit and shadow areas in each rocky outcrop category using DPM. The predicted cover in sunlit areas was 5% to 15% smaller than reference values in areas with rocky outcrop cover less than 90%. However, we found the opposite result in shadow areas where predicted values were 15% to 54% larger than reference data. Therefore, the percent of rocky outcrop bedrock was underestimated in sunlit and overestimated in shadow areas as predicted by DPM. MESMA, SESMA and DPM predicted that the rocky outcrop cover of 40.8%, 28.4% and 36.6% respectively across the study area.

The standard error (SE) of predicted cover was distributed between 1% and 20% using all estimate methods (Fig. 2). SE from MESMA and SESMA showed similar trends in each class of predicted cover with low SE values (5–12%) in most rocky cover areas. The pattern differed where rocky cover was highest. These low values indicated that the predicted cover of rocky outcrop is accurate. In general, the SE in the sunlit areas was lower than that in shadow areas using these two methods, indicating greater accuracy in sunlit than in shadow areas. DPM had higher SE compared with the other two SMA methods suggesting that the value of predicted cover distributed discretely compared with referenced data.

**Figure 3:**
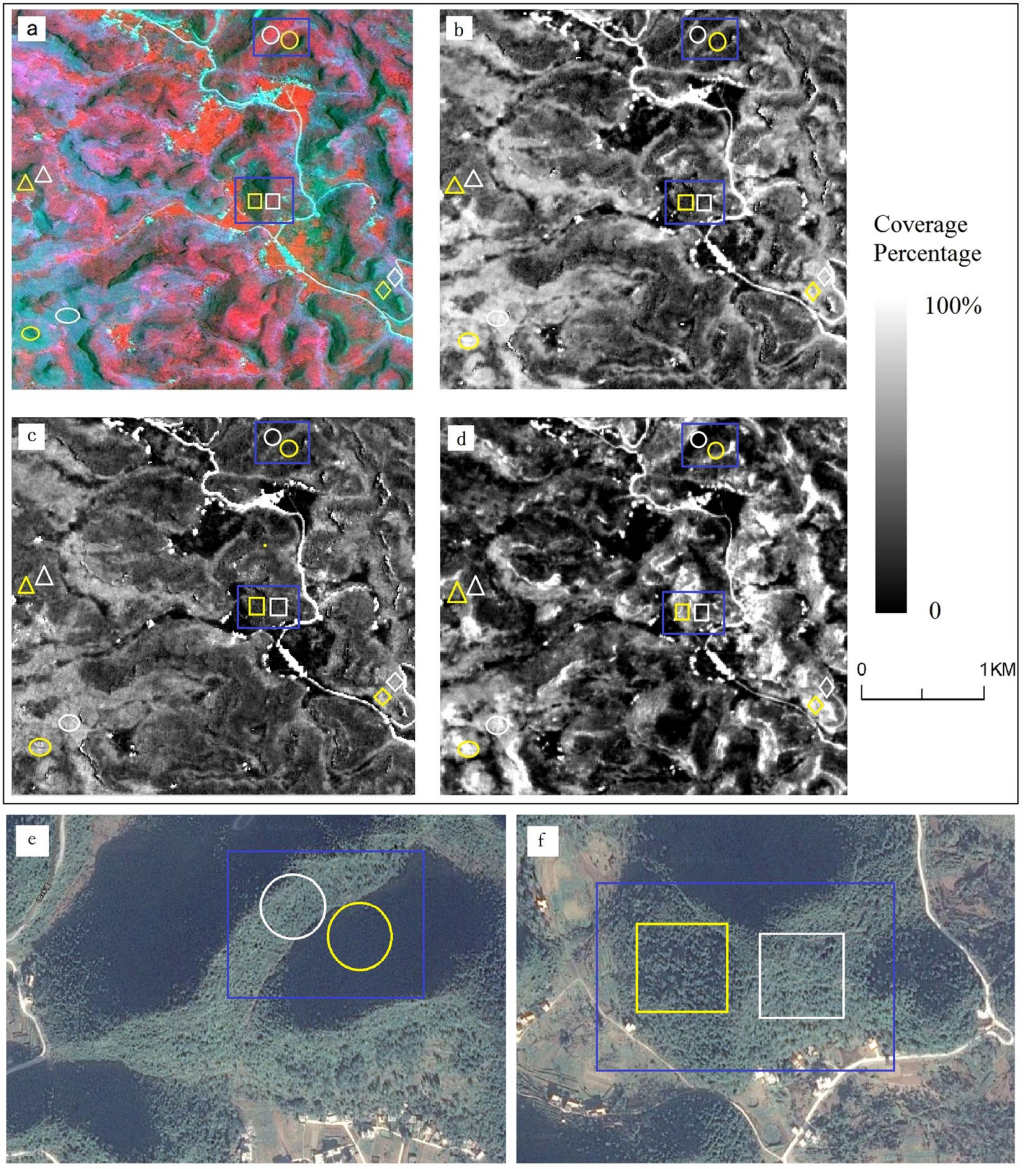
Contrast of rocky outcrop cover in sunlit and shadow areas. Panel a is an ALOS sharpened images (RGB: NIR, red, and green bands). Panels b–d are fraction rock cover predicted using MESMA, SESMA and DPM respectively. Circle, square, triangle, diamond and ellipse symbols represent the rock coverage less than 10%, 20%, 30–40%, 55–70% and 75% respectively. Symbols in white are in sunlit areas and those in yellow are in shadow. Panels *e* and *f* show identical land covers where only the aspects are different in sunlit and shadow areas. These two image clips are extracted from satellite imagery (acquired on October 1, 2012) available on Google Earth and data provider is Digital Globe.

**Figure 4:**
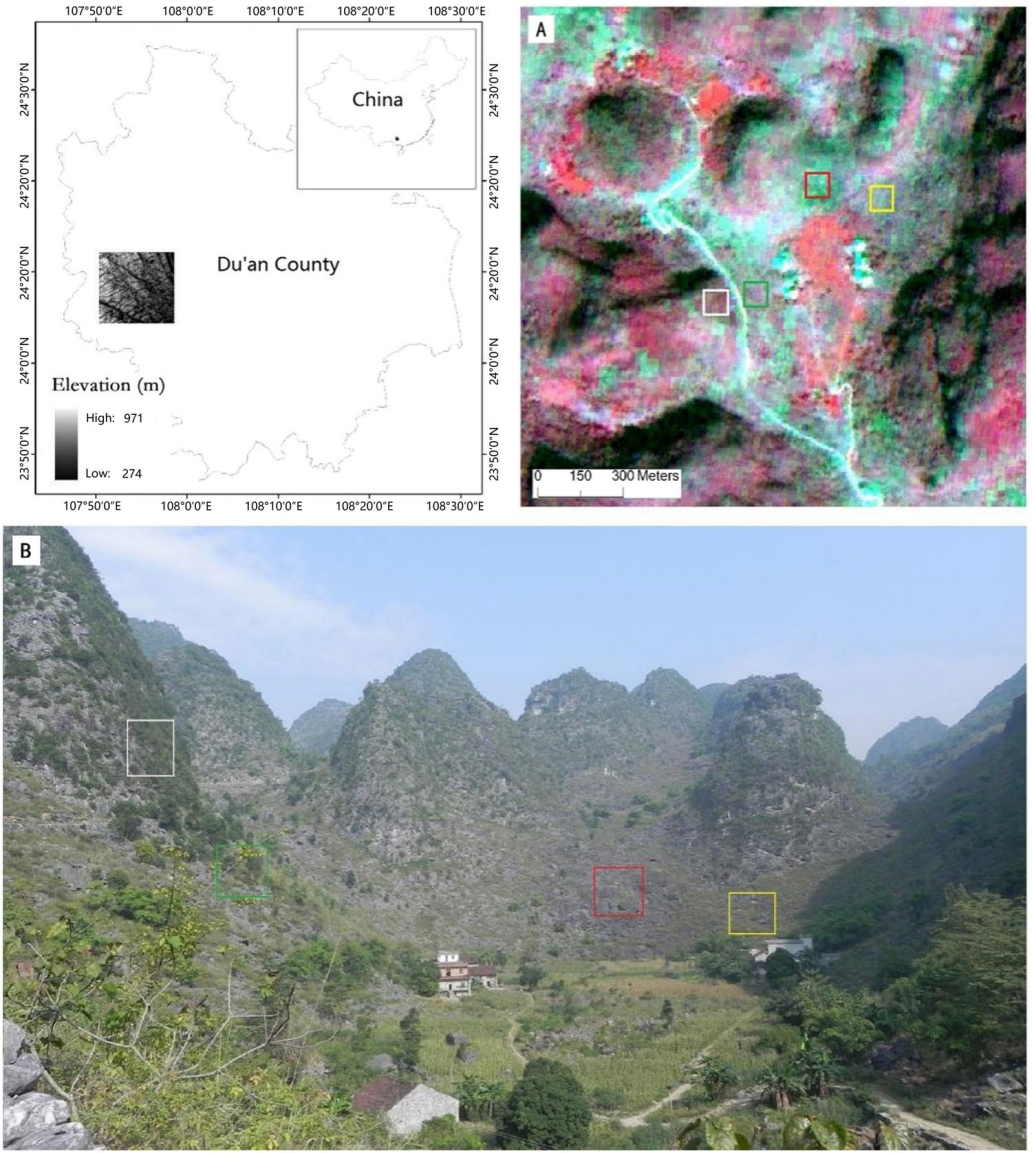
Study area and characteristics of KRD. Panel A is a pan-sharpened ALOS image (RGB: NIR, red, and green). This sharpened image is located in study area (**B**). Panel (B) is a photograph taken during the field survey. Red, yellow, green and white squares represent reference sites with severe, moderate, light and potential KRD respectively.

Shadow effects made similar land features appear differently by varying the spectra in sunlit and shadow areas on satellite images (Fig. 3a). Compared with the original satellite images, fractions from MESMA and SESMA had few spectral changes and therefore had similar grey values for similar land types in sunlit areas (white border) and shadow area (yellow border) (Fig. 3b,c). This indicated that the rocky fractions predicted by MESMA and SESMA might reduce shadow effects. Compared with the images shown in Fig. 4b,c, the rocky fraction from DPM had a shadow effect demonstrated by a large difference in white and yellow bounded areas (Fig. 3d). We used the cosine of incident angle that was calculated based on DEM data to represent the shadow effect of satellite images. The lower the correlation coefficient between the rocky fraction and the cosine of incident angle, the smaller the impact of the shadow^33^. This shows that the percentage of rocky fractions from MESMA and SESMA had low correlation coefficients with the cosine of incident angle (0.02 and 0.01, respectively; P < 0.01). Contrast this with the rocky fraction of DPM and cosine of incident angle that had a relatively high correlation coefficient (0.33; P < 0.01). A higher regression coefficient means the measured rocky fraction from DPM was affected by terrain relief. Estimations from MESMA and SESMA of the percent of rocky outcrops could reduce the topographic effects in satellite images by shade normalization.

### KRD mapping

Based on MESMA, SESMA and DPM methods, the summed areas of light, moderate and severe KRD covered 62.7%, 39.5% and 56.5% of the total area, respectively (Table 3). Using MESMA, the non KRD, potential KRD, light KRD, moderate KRD and severe KRD covered 6.8%, 30.5%, 34.0%, 20.6% and 8.1% of the study area, respectively. Potential, light and medium moderate KRD dominated the region. The main KRD type predicted by SESMA was potential KRD that covered 53.8% of the study area. Given that the study area is typical of KRD affected regions where vegetation degradation, severe soil erosion and exposed rock phenomena are common^15^, KRD areas appeared to be underestimated by SESMA. Although the total area of KRD from DPM seemed reasonable, its distribution was affected by terrain relief. This appeared to be because rocky outcrop cover underestimation in sunlit areas corresponded to the distribution of potential and light KRD. In contrast, rocky coverage overestimation in shadow areas largely correlated to the moderate and severe KRD distribution.

**Table 3:** Percent area at each KRD level based on different methods.

KRD percentage (%)	non-KRD	potential KRD	light KRD	moderate KRD	severe KRD
MESMA	6.8	30.5	34.0	20.6	8.1
SESMA	6.7	53.8	33.5	2.5	3.5
DPM	15.6	27.9	25.7	16.3	14.5

## Discussion

Rocky desertification monitoring is an important task for environmental management of southwest China. Previous research on remote sensing of KRD estimated the percentage of rocky outcrop cover using DPM^13^. Our results suggest that fractional rocky cover was underestimated by 5% to 15% in sunlit areas based on new methods. This could be the result of nonlinearity of NDVI; NDVI tends to be sensitive to sparse vegetation cover^36,37^. Therefore, the fractional vegetation cover may be overestimated in sunlit areas with an accordingly underestimated coverage of rocky outcrops.

The high heterogeneity of the karst landscape poses a within-pixel mixing problem for remote sensing information extraction. SMA appears to be a feasible resolution^20,29^. Although there are no studies that use MESMA-based fraction images to estimate KRD levels, SMA has proven to be efficient in detecting a signal from impervious surfaces in urban areas^31^. Unlike the regularity of urban impervious surfaces however, features of rocky outcrops show a degree of surface variability. Due to differences in human disturbance intensity, degree of weathering and erosion, rocky outcrop surface colors may vary. Field investigations have shown that bedrock outcrops are often white-gray resembling human ploughed soil, mined mineral or clipped vegetation. Especially in mined areas, rocky outcrops had high spectral reflectance on cut surfaces (Fig. 1a; a spectral curve of bedrock with high values). When natural forces, like weathering or dissolution, dominated surface bedrock, outcrops appeared gray. Color differences among rocky outcrop surfaces may cause variation in spectral reflectance (Fig. 1b).

Using SESMA, we selected one optimal endmember for three searched land cover types from a vertex of spectral scatter plots^31^. This approach only allowed one spectrum as a pure pixel for each endmember. In our study, the spectra of rocky outcrops from SESMA had higher reflectance compared with those from MESMA (Fig. 1). If this one optimal endmember effectively represents rocky outcrops, the fractional cover would be estimated more accurately. Otherwise, the spectrum only represents parts of bedrock features (like cut surfaces) and the fractional cover would be routinely underestimated. According to the error matrix of accuracy assessment, results from SESMA underestimated 1% to 27% of the fractional cover of rocky outcrops. Many areas dominated by rocky outcrops were classified as low or moderate rocky covered area. This may have been caused by SESMA’s optimal spectral curve not representing the general features of rocky outcrops in karst areas. For that reason, an optimal endmember collection based on the SESMA model is difficult to incorporate the spectral variation of feature classes in the karst region. The underestimation of SESMA predicted rocky outcrop coverage of the whole study area to be 28.4%. This did not match the study area reality land types with light and moderate KRD impacts^15,18,38^.

MESMA extends SESMA by allowing the number and type of endmembers to vary on a per-pixel basis^26^. This overcomes SESMA’s limitations by requiring a model to meet minimum fit, fraction and residual constraints while testing multiple models for each image pixel. The optimal endmembers from MESMA were selected through calculating the lowest RMSE within a class^35^. MESMA allows a certain number of optimal endmembers to be selected from images. When one land cover has variable spectral reflectance, it is better to have many representative spectral curves to capture its features. Indeed, both natural factors and human activities have contributed to instances of KRD, contributing to image variability^13,18^. This diversity of factors results in differences in the appearance of exposed bedrock. Nevertheless, our accuracy assessment results showed that much of rocky outcrop cover could be predicted accurately. Therefore, multiple endmembers are indeed more reliable than single endmembers in applying karst exposed rock monitoring.

A possible shortcoming in our optimal endmember selection is the number of endmembers needed. Although our study masked built-up and water areas and used remotely sensed images from the crop-growing season so that the bare soil would be covered by vegetation, there was still some bare soil. KRD is, by definition, the landscape of exposed bedrock after the soil is lost^5^. Rocky outcrops appear as ragged cover accompanied by numerous rock fissures after weathering and soil erosion. There is often some soil, deciduous plants or bryophytes in those rock cracks^18^. Because of the restricted number of bands in ALOS images, only three types of endmembers were suitable for selecting to model fraction image. Field-based spectra of the karst land surface showed that non-photosynthetic vegetation, bare soil and rocky outcrops had similar spectral responses at 350–350  nm, and these differences were mainly focused at 1350–1350 nm^39,40^. Therefore, bare soil might be confused with exposed rock in SMA modeling in our study. In future studies, imagery with more spectral bands (such as WorldView-3, that has 8 bands) should be used to enable both the selection of more endmembers and improve the accuracy of remote sensing information extraction in highly heterogeneous regions.

In karst regions, rugged terrain is oriented by the development of carbonate rock. Steep elevation change is common. Rugged terrain not only affects vegetation growth but also affects the extraction of vegetation information^34^. The spectral reflectance of land cover is often strongly affected by terrain relief in medium and high spatial resolution satellite imagery. In our study this caused variation in DN values in sunlit and shadow areas. Previous studies have shown that a topographic correction model does not effectively improve the classification accuracy of the ground objects^13,41,42^. Furthermore, there are few studies where the spectral differences of the same land objects are compared between sunlit and shadow areas in a karst region after topographic correction.

The results of our study showed that estimated rocky outcrop coverage from SESMA and MESMA was similar in sunlit and shadow areas, largely because the shade fraction, as an independent component, was extracted by least squares in the SMA model and removed through shade normalization^43^. In fact, shadows from mountains, tall trees, and even protruding stones, commonly occur in karst regions. However, previous studies have paid less attention to shadow and its impacts on remote sensing applications in karst areas^33^. One of the advantages of SMA model is that it accounts for shade. These results demonstrate that SMA provides a new perspective for topographic correction of remote sensing applications in a mountainous region.

DPM, as a simple and effective method for managing topographic effects, is widely used to monitor vegetation cover^32,44^. However, the DPM-predicted vegetation cover between sunlit and shadow areas was very different in our study. The reason for this appears to be that, when the aspect transformed from sun to shadow, the DN value of the near infrared band decreased more rapidly than the red band (Fig. 1). Based on the DPM formula, the predicted value of rocky outcrop cover increases in shadow. The differences between sunlit and shadow areas caused by topographic effects influenced the accurate inversion of land surface information of KRD and further affected KRD mapping. Therefore, topographic effects based on DPM could not be ignored when using optical remotely sensed monitoring in this mountainous region.

## Conclusions

With the objective of overcoming obstacles to monitoring heterogeneous, rugged terrain on rocky deserts using remote sensing, our study applied high spatial resolution ALOS images and compared DPM, SESMA and MESMA to extract the key indicators of KRD at a sub-pixel scale. The optimization results of accuracy assessment were acquired using MESMA. The overall accuracy in the sunlit and shadow areas were 83.7% and 60.4%, respectively. The SESMA approach attained lower accuracy because it underestimated between 1% and 25.7% of the fractional cover of rocky outcrops. However, mean coverage of the same objects was similar in sunlit and shadow areas although the accuracy in the shadow was lower based on these two SMA methods. Correlation analysis and spatial statistics demonstrated that SMA methods could reduce shadow effects on fractional cover extraction in karst regions.

Shadows that came from mountains, tall trees or raised rocks, were widespread and shadow effects were one of the critical factors affecting classification accuracy of land use and land cover in remote sensing applications in the karst region of southwestern China. One of the advantages of the SMA model is that it accounts for the shadow fraction and can remove its influence. We concluded that SMA provides a new perspective on topographic correction for remote sensing applications in mountainous regions. KRD distribution can be mapped accurately with the fractional estimation by MESMA. Approaches cannot ignore that the fractional cover of rocky outcrops may be underestimated in the sunlit areas and overestimated in shadow areas when using DPM methods. Therefore, the topographic impacts on NDVI could not be overlooked in karst vegetation monitoring.

## Materials and Methods

### Study area

Our study area is in Du’an, Guangxi Province, southwestern China. The topography is rugged with elevations between 230 and 1050 m. Typical landforms in this region are tower karsts and depressions. Although the climatic vegetation climax community in this area is subtropical evergreen forest, dominant vegetation communities are grass and shrub because of severe human disturbance (Fig. 4).

**Figure 5:**
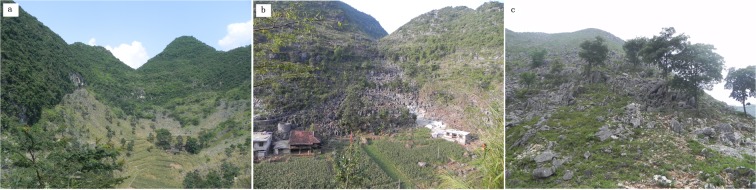
Typical landscapes of rocky desertification in the study area. The rocky desertification in panels a and b were the result of cultivation on steep slopes. Panel c shows the consequence of firewood harvesting where only a few trees remained.

This area has a relatively high population density (52 people per km^2^ in 2005) and a >1000-year history of agricultural development. Timber and firewood harvesting and intensive agricultural practices on the slopes have led to the disappearance of the climax community in this region during the 1950s to the 1980s. By the 1990s, 60% to 70% of the forested area in the karst regions was cleared, and most existing forests were early seral, secondary vegetation^45^ (Fig. 5). Severe human disturbance and soil erosion also caused slow vegetation recovery. The coexistence of rocky outcrops and vegetation cover at a fine scale is common in the karst region.

### Data and preprocessing

Advanced Land Observing Satellite (ALOS) imagery was acquired on June 4th, 2009. These data contained four multispectral bands commonly used in remote sensing studies (blue, green, red and near infrared) with a 10 m spatial resolution and a panchromatic band with 2.5 m spatial resolution. We choose multispectral ALOS imagery for KRD information extraction at the sub-pixel scale. The solar elevation and azimuth of this image is 73.3° and 93.8° respectively. Although the relatively acute solar elevation reduces shadow effects on images^41^, there was still a high percentage (about 25% of total area in this study) of shadow in images (Fig. 4A). Images were processed using level L1G systematic correction.

Atmospheric correction of multispectral ALOS imagery was conducted using the MODTRAN5 model. We georeferenced images and projected them to a Universal Transverse Mercator (UTM) map projection. The original multispectral and panchromatic images were fused by the Gram–Schmidt procedure in the ENVI software package^46^ to produce four-band, pan-sharpened multispectral ALOS images. A matched digital elevation model (DEM) was applied to calculate the cosine of solar incident angle as quantified shadow effects of ALOS imagery^33,42^.

### Dimidiate pixel model

DPM was used to calculate fractional vegetation cover (FVC)^47^. The percentage of rocky outcrops equals one minus the FVC value as soil background was covered by crops. The Normalized Difference Vegetation Index (NDVI) was applied:

1 $$NDVI=({\rho }_{{\rm{nir}}}-{\rho }_{{\rm{red}}})/({\rho }_{{\rm{nir}}}+{\rho }_{{\rm{red}}})$$

where ρ_nir_ and ρ_red_ are the surface reflectance of near infrared and red bands respectively. It has been demonstrated that FVC follows a linear relationship with the NDVI:

2 $${F}_{DPM}=(NDVI-NDV{I}_{S})/(NDV{I}_{V}-NDV{I}_{S})$$

where *NDVIs* and *NDVIv* are representative values of NDVI for bare rock pixels (where *F*_*DPM*_ = 0) and 100% vegetated pixel (*F*_*DPM*_ = 1) respectively. The calculation of DPM was implemented with ITT ENVI software.

### Spectral unmixing models

Simple endmember liner spectral mixture analysis (SESMA) and multi-endmember spectral mixture analysis (MESMA) were applied to extract bedrock cover. SESMA extracts only one optimal endmember for each independent class. MESMA expands the numbers and focuses on selecting one or several representative endmembers within each land cover type. Both SESMA and MESMA assume that the spectrum measured by a sensor is a linear combination of the spectra of all components within the pixel^48^. The mathematical model can be expressed as:

3 $${R}_{i}={\sum }_{{\rm{k}}=1}^{n}\,{f}_{k}{R}_{ik}+{\varepsilon }_{i}$$

where *i* is the number of spectral bands used, *k* is the number of endmembers 1, …, *n*, R_*i*_ is the spectral reflectance of band *i* of a pixel that contains one or more endmembers, *f*_*k*_ is the proportion of endmember *k* within the pixel, R_*ik*_ is the spectral reflectance of endmember *k* within the pixel on band *i*, and *ε*_*i*_ is the error for band *i*. A common approach for obtaining *f*_*k*_ is to use a least-squares solution by minimizing the residual error with the sum of *f*_*k*_ of all optical endmembers equal to one. The spectral mixture analysis consists of three primary steps: (1) selection of candidate endmembers to build a spectral library, (2) optimal endmember selection, and (3) decomposing the mixed pixels to extract fractional images.

We applied the vegetation-high albedo-shadow model that was found to be most suitable for non-urban areas to extract remotely sensed information^49^. Vegetation endmembers included grass, shrub, forest and crops in the karst region. Ground features with high albedo include rocky outcrops, road surfaces, and rooftops. Low albedo features include shadow and water. As only three endmembers could be selected for model inputs, road surfaces and rooftops were masked identified by their reflectance in the red band. Their values are generally higher than that of rocky outcrops based on field surveys. Water areas were also masked using the ratio of green and near infrared bands^50^. Consequently, other features, including vegetation, rocky outcrops and shadow, were kept for SMA modeling. We used a spectral scatter plot to select candidate endmembers. The scatter plot triangulated with the three vertices representing vegetation, rocky outcrop and shadow. Finally, 291, 185 and 159 candidate endmembers for the three land types (vegetation, rocky outcrop and shadow) were identified for further processing.

Identifying high quality image endmembers has been described as a critical stage of spectral mixture modeling^51^. We initially selected optimal endmembers on images according to field survey sites to compare with two other methods. The SESMA method is a fully-constrained linear spectral unmixing method based on a high-vegetation-high albedo-low albedo model^31^. The spectral scatter plots were generated as a triangle (here defined as ΔOAB). The best three endmembers could be identified by the largest area (S) among all triangles formed by any three pixels:

4 $$S=|\overrightarrow{{\rm{a}}}\times \overrightarrow{{\rm{b}}}|/2$$

where $\overrightarrow{{\rm{a}}}$ and $\overrightarrow{{\rm{b}}}$ denote the endmember vectors of OA and OB, respectively. The detailed calculation of SESMA in this research follows Yang^31^.

For the MESMA method, an endmember average RMSE (root mean squared error) (EAR) approach was used to select the most appropriate endmembers. The endmembers were selected by calculating the lowest RMSE within a class^31^. EAR was calculated using Eq. (4) using:

5 $$EA{R}_{i}=({\sum }_{i=1}^{N}RMS{E}_{i,j})/(n-1)$$

where *i* is an endmember, *j* is the modeled spectrum, *N* is the number of endmembers, and *n* is the number of modeled spectra. The “−1” corrects for the zero-error resulting from an endmember model itself. Here, eight endmembers of vegetation, rocky outcrop and shadow were selected according to the lowest RMSE. MESMA in this study was implemented with VIPER Tools, a plug-in software under ITT ENVI^26,31^.

Fractional maps of endmember land cover components were generated by optimal endmember models using least-squares solutions. To reduce shadow effects, we performed a shade normalization of the fraction images obtained by dividing the cover of each endmember by the total percent cover of all non-shade endmembers (1 minus shade fraction) in each pixel. This suppresses the shade fraction so that we obtain more information from the other two fractions (vegetation and rock)^43^.

### Accuracy assessment

Accuracy assessments for rocky outcrop coverage were conducted using error matrices. A Kappa coefficient was used to measure the accuracy of the predicted rocky outcrop coverage^52^. Overall accuracy (OA) for each class was also calculated. The percentage of rocky outcrop was classified into five categories (0–10%, 10–30%, 30–50%, 50–70% and 70–100%, which generally represent, in order, non-KRD, potential KRD, light KRD, moderate KRD and severe KRD) using the suggested threshold values for KRD assessment^53^.

Field validation sites were sampled from 2009 to 2011 in Du’an county. Each site was 30 × 30 m. Transect sampling methods^54^, visual observations and photograph interpretation were combined to estimate the fractional cover of vegetation and rocky outcrop at each site. The measured sample sites were principally located near a road or path as large-relief mountain areas are difficult to access. Most validation sites for accuracy assessments were determined by the coordination of visual estimates of rocky outcrop cover during field visits. Images from Google Earth were also used to validate land types in shadow. Finally, 539 validation points (159 of which were in shadow) were collected to verify the accuracy of the fractional cover estimates from the SESMA, MESMA and DPM with ALOS data.

### KRD mapping

Based on the accuracy assessment results, an estimate of severity levels (non-KRD, potential, light, moderate and severe KRD) was achieved by classifying the optimal fractional cover of rocky outcrops using a decision tree classifier. Rules for the decision tree were established by expert knowledge and C5.0 decision tree algorithms. The threshold value of KRD levels referenced previous studies^15,38,53^ (see above).

## References

[1] Legrand HE (1973). Hydrological and ecological problems of karst regions. Science..

[2] Parise M, Gunn J (2007). Natural and anthropogenic hazards in karst areas: recognition, analysis and mitigation. J. Geol. Soc. Lond..

[3] Su W (2002). Controlling model for rocky desertification of karst mountainous region and its preventing strategy in southwest China. J Soil Water Conserv..

[4] Cai YL (1996). Preliminary research on ecological reconstruction in karst mountain poverty areas of southwest China. Adv. Earth Sci..

[5] Yuan, D. X. *The Karstkarst study of China*. Geology Press Beijing (1993).

[6] Zhang MY (2010). Using the radial basis function network model to assess rocky desertification in northwest Guangxi, China. Environ. Earth Sci..

[7] Chinese Academy of Sciences (2003). Several suggestions for the comprehensive taming to karst mountain areas in southwest China. Bull. Chin. Acad. Geol. Sci..

[8] Song CH, Schroeder TA, Cohen WB (2007). Predicting temperate conifer forest successional stage distributions with multitemporal Landsat Thematic Mapper imagery. Remote Sens. Environ..

[9] Mello AYI, Alves DS (2011). Secondary vegetation dynamics in the Brazilian Amazon based on thematic mapper imagery. Remote Sens. Letters..

[10] Xiao J, Moody A (2005). A comparison of methods for estimating fractional green vegetation cover within a desert-to-upland transition zone in central New Mexico, USA. Remote Sens. Environ..

[11] Diao, S. J. & Nie, H. F. Remote sensing technology and western development. *Remote Sens*. *Land Resour*. **4**, 7–12 (2000). (In Chinese with English abstract).

[12] Ahmady-Birgani H, McQueen KG, Moeinaddini M, Naseri H (2017). Sand dune encroachment and desertification processes of the Rigboland Sand Sea, Central Iran. Sci. Rep..

[13] Zhang CH, Qi XK, Wang KL, Zhang MY, Yue YM (2017). The application of geospatial techniques in monitoring karst vegetation recovery in southwest China: A review. Prog. Phys. Geos..

[14] Huang QH, Cai YL (2007). Spatial pattern of karst rock desertification in the middle of Guizhou province, southwestern China. Environ. Geol..

[15] Yang QQ, Wang KL, Zhang CH, Yue YM, Tian RC (2011). Spatio-temporal evolution of rocky desertification and its driving forces in karst areas of Northwestern Guangxi, China. Environ. Earth Sci..

[16] Bai XY, Wang SJ, Xiong KN (2013). Assessing spatial-temporal evolution processes of karst rocky desertification land: indications for restoration strategies. Land Degrad. Dev..

[17] Du H, Hu F, Zeng F, Wang K, Song T (2017). Spatial distribution of tree species in evergreen-deciduous broadleaf karst forests in southwest China. Sci. Rep..

[18] Yue YM (2013). Development of new remote sensing methods for mapping green vegetation and exposed bedrock fractions within heterogeneous landscapes. Int. J. Remote Sens..

[19] Pu RL, Gong P, Michishita R (2008). Spectral Mixture Analysis for Mapping Abundance of Urban Surface Components from the Terra/ASTER Data. Remote Sens. Environ..

[20] Adams, J. B. & Gillespie, A. R. Remote sensing of landscapes with spectral images: A physical modeling approach. Cambridge University Press (2006).

[21] Smith, M. O., Adams, J. B. & Sabol, D. E. *Spectral mixture analysis-new strategies for the analysis of multispectral data. In Imaging spectrometry—a tool for environmental observations*. Springer, Dordrecht. 125–143 (1994).

[22] Jiang MC, Tian S, Zheng Z, Zhan Q, He Y (2017). Human Activity Influences on Vegetation Cover Changes in Beijing, China, from 2000 to 2015. Remote Sens..

[23] Wang MC, Wang XZ, Liang ZX (2014). Landscape pattern analysis on change of fractional vegetation cover between karst and no-karst areas: A case study in Hechi District, Guangxi Zhuang Autonomous Region. Acta Ecol. Sin..

[24] Xu J, An YL, Hu F (2015). Research on characteristics of ecological environment in a semi-karst region based on vegetation cover and NPP: A case study in central Guizhou province. Geogr. Res..

[25] Somers B, Asner GP, Tits L, Coppin P (2011). Endmember variability in spectral mixture analysis: A review. Remote Sens. Environ..

[26] Roberts DA (1998). Mapping chaparral in the Santa Monica Mountains using Multiple Endmember Spectral Mixture models. Remote Sens. Environ..

[27] Youngentob KN (2011). Mapping two Eucalyptus subgenera using multiple endmember spectral mixture analysis and continuum-removed imaging spectrometry data. Remote Sens. Environ..

[28] Eckmann TC, Roberts DA, Still CJ (2009). Estimating subpixel fire sizes and temperatures from ASTER using Multiple Endmember Spectral Mixture Analysis. Int. J. Remote Sens..

[29] Quintano C, Fernández-Manso A, Roberts DA (2013). Multiple Endmember Spectral Mixture Analysis (MESMA) to map burn severity levels from Landsat images in Mediterranean countries. Remote Sens. Environ..

[30] Franke J, Roberts DA, Halligan K, Menz G (2009). Hierarchical Multiple Endmember Spectral Mixture Analysis (MESMA) of hyperspectral imagery for urban environments. Remote Sens. Environ..

[31] Yang J, He YH (2017). Automated mapping of impervious surfaces in urban and suburban areas: Linear spectral unmixing of high spatial resolution imagery. Int. J. Appl Earth Obs..

[32] Huang QH, Cai YL (2009). Mapping Karst Rock in Southwest China. Mt. Res. Dev..

[33] Qi XK, Wang KL, Zhang CH (2013). Effectiveness of ecological restoration projects in a karst region of southwest China assessed using vegetation succession mapping. Ecol. Eng..

[34] Tong XW (2016). Assessing Future Vegetation Trends and Restoration Prospects in the Karst Regions of Southwest China. Remote Sens..

[35] Dennison PE, Roberts DA (2003). Endmember selection for mapping chaparral species and fraction using Multiple Endmember Spectral Mixture Analysis. Remote Sens. Environ..

[36] Jiang Z (2006). Analysis of NDVI and scaled difference vegetation index retrievals of vegetation fraction. Remote Sens. Environ..

[37] Obata K, Miura T, Yoshioka H (2012). Analysis of the scaling effects in the area-averaged fraction of vegetation cover retrieved using an NDVI-isoline-based linear mixture model. Remote Sens..

[38] Xu EQ, Zhang HQ, Li MX (2015). Object-Based Mapping of Karst Rocky Desertification using a Support Vector Machine. Land Degrad. Dev..

[39] Fu BH (1996). A Study on Reflectance Spectra Features of Carbonate Rocks and Its Application. Rock Miner. Anal..

[40] Yue YM (2010). Spectral Indices for Estimating Ecological Indicators of Karst Rocky Desertification. Int. J. Remote Sens..

[41] Song CH, Woodcock CE (2003). Monitoring forest succession with multitemporal Landsat images: Factors of uncertainty. IEEE Trans. Geosci. Remote Sens..

[42] Gao YN, Zhang WC (2009). A simple empirical topographic correction method for ETM plus imagery. Int. J. Remote Sens..

[43] Rogan J, Franklin J (2001). Mapping wildfire burn severity in Southern California forests and shrublands using enhanced Thematic Mapper imagery. Geocarto Int..

[44] Helmer EH, Brown S, Cohen WB (2000). Mapping montane tropical forest successional stage and land use with multi-date Landsat imagery. Int. J. Remote Sens..

[45] Wen, L. *et al*. The succession charactersitcs and its driving mechanism of plant community in karst region, southwest China. *Acta Ecol*. *Sin*. **35**, 5822–5833 (2015). (In Chinese with English abstract).

[46] Laben, C. A. & Brower, B. V. Process for enhancing the spatial resolution of multi-spectral imagery using pansharpening. U.S. Patent No. 6,011,875, Eastman Kodak Company (2000).

[47] Gutman G, Ignatov A (1998). The derivation of the green vegetation fraction from NOAA/AVHRR data for use in numerical weather prediction models. Int. J. Remote Sens..

[48] Adams JB, Smith MO, Gillespie AR (1993). Imaging spectroscopy: Interpretation based on spectral mixture analysis. Remote geochemical analysis: Elemental and mineralogical composition..

[49] Xiao JF, Moody A (2005). A comparison of methods for estimating fractional green vegetation cover within a desert-to-upland transition zone in central New Mexico, USA. Remote Sens. Environ..

[50] McFeeters SK (1996). The use of the Normalized Difference Water Index (NDWI) in the delineation of open water features. Int. J. Remote Sens..

[51] Tompkins S, Mustard JF, Pieters CM, Forsyth DW (1997). Optimization of endmembers for Spectral Mixture Analysis. Remote Sens. Environ..

[52] Congalton, R. G. & Green, K. *Assessing the accuracy of remotely sensed data Principles and practices* (2nd ed.). Boca Raton: CRC Press. Taylor & Francis. (2009).

[53] Li YB, Shao JA, Yang H, Bai XY (2009). The relations between land use and karst rocky desertification in a typical karst area, China. Environ. Geol..

[54] Delameter PL, Messina JP, Qi JG, Cochrane MA (2012). A Hybrid Visual Estimation Method for the Collection of Ground Truth Fractional Coverage Data in a Humid Tropical Environment. Int. J. Appl Earth Obs..

